# A Multi-Sensor, Multi-Movement Exploratory Study of Motion Tape Strain Data for Low Back Pain Classification

**DOI:** 10.3390/s26134187

**Published:** 2026-07-02

**Authors:** Pratham Yashwante, Sara P. Gombatto, Yasmín Velázquez, Elijah Wyckoff, Aarti Lalwani, Kevin Patrick, Kenneth J. Loh, Emilia Farcas, Rose Yu

**Affiliations:** 1 Computer Science and Engineering, University of California San Diego, La Jolla, CA 92093, USA; pyashwante@ucsd.edu (P.Y.); adlalwani@ucsd.edu (A.L.); roseyu@ucsd.edu (R.Y.); 2 School of Physical Therapy, San Diego State University, San Diego, CA 92182, USA; 3 School of Exercise and Nutritional Sciences, San Diego State University, San Diego, CA 92182, USA; yvelazquez3@sdsu.edu; 4 Active, Responsive, Multifunctional, and Ordered-materials Research (ARMOR) Laboratory, Department of Structural Engineering, University of California San Diego, La Jolla, CA 92093, USA; ewyckoff@ucsd.edu (E.W.); kenloh@ucsd.edu (K.J.L.); 5 Qualcomm Institute, University of California San Diego, La Jolla, CA 92093, USA; kpatrick@ucsd.edu (K.P.); efarcas@ucsd.edu (E.F.); 6 School of Public Health, University of California San Diego, La Jolla, CA 92093, USA

**Keywords:** biomechanical sensing, functional movement, low back pain, strain-based features, time-series analysis, wearable sensors

## Abstract

Objective assessment of low back pain (LBP) is challenging due to subtle, task-dependent movement impairments that are poorly captured by existing sensing technologies. Motion Tape (MT), which is a self-adhesive elastic fabric skin strain sensor, enables skin-conforming measurement of localized biomechanical strain during functional movement, but its discriminative utility for LBP remains unclear. We examine this question in a multi-sensor, multi-movement setting and analyze whether MT signals encode discriminative structure that distinguishes individuals with LBP from healthy controls. Using data from 20 participants performing 19 functional movements with six sensors, we evaluate movement-specific classification under a leave-pair-out protocol and examine which movements, sensor placements, and features are most informative. Our analysis reveals that group separation is highly selective: only a small subset of movements, most notably forward flexion, consistently supports accurate classification, while many movements remain at near-chance level. We find that temporal dynamics features help in resolving difficult cases that global strain statistics fail to separate, and that informative signals are spatially localized to the lower lumbar spine. In contrast, pretrained time-series foundation models show negligible sensitivity to participant-level structure in MT signals. Overall, the findings from this exploratory study establish when and how MT sensing can effectively differentiate individuals with LBP from healthy controls, providing a principled foundation for larger-scale validation.

## 1. Introduction

Low back pain (LBP), defined as pain localized between the lower rib margin and gluteal folds, is among the most prevalent and burdensome health conditions worldwide [1,2,3]. In 2020 alone, over 619 million individuals were living with LBP globally, resulting in more than 69 million cumulative years of healthy life lost due to disability, and with prevalence projected to rise to over 843 million cases by 2050 [4]. In addition, LBP imposes substantial economic and societal costs, exceeding 100 billion dollars annually in the United States [5] and approximately 149.1 million lost workdays each year [6]. Despite its prevalence, the etiology of LBP remains poorly understood; in nearly 85% of cases, no specific structural cause can be identified, and correlations between pain symptoms and imaging findings are weak [7,8].

### 1.1. Background on Biomechanics of LBP

A growing body of evidence suggests that non-specific LBP is associated with altered patterns of movement and muscle engagement [9,10,11,12]. Individuals with LBP often exhibit reduced range of motion, altered trunk muscle activation, impaired proprioception, and slower or more variable movement strategies compared to healthy controls (CTRL) [9,11,12,13,14]. Beyond peripheral biomechanics, chronic LBP is also associated with reorganization at cortical, subcortical, and spinal levels, which may further influence movement patterns [15]. Segmental lumbar kinematics measured via optical motion capture have shown that lower lumbar levels exhibit greater range of motion during diagnostic movements like flexion and box lifting, and that such baseline data from healthy cohorts can inform comparisons for pathological movement patterns [16]. These biomechanical changes are particularly relevant in physical therapy, where clinicians rely heavily on the observation of posture and movement to assess impairments, guide interventions, and monitor the return phase of the movement. However, objective and scalable tools for measuring lower back movement and muscle engagement, especially outside of laboratory environments remain limited.

### 1.2. Existing Sensing Technologies and Usage of Machine Learning

Current technologies for assessing low back posture and movement each have notable limitations. Optical motion capture systems are considered the gold standard for biomechanical human motion analysis but are expensive, require specialized expertise, and are confined to laboratory settings [17,18,19]. Inertial measurement units provide portable alternatives but suffer from rigid form factors, sensitivity to sensor placement and drift, and limited ability to capture multi-segmental spinal motion or skin deformation [19,20,21,22]. Electromyography measures muscle activation effectively but is costly and poorly suited for free-living monitoring, limiting its use for longitudinal or remote assessment of LBP-related muscle engagement [23,24,25]. Related work using inertial sensors and motion capture has also demonstrated that machine learning can identify chronic LBP patients from instrumented trunk bending tasks, classify pathological range of motion, and predict pain-related outcomes from lifting techniques, achieving high accuracy [26,27,28].

### 1.3. Motion Tape

Motion Tape (MT) is an emerging wearable sensing technology designed to address many of these limitations. MT is fabricated by depositing graphene nanosheets [29,30] or carbon nanotubes [31] onto commercial kinesiology tape, forming a flexible, piezoresistive strain sensor that conforms naturally to the body. Fabrication begins by spray-coating or drop-casting the nanomaterial dispersion directly onto commercially available kinesiology tape to form a rectangular fiber-integrated nanocomposite [32]. Kinesiology tape is employed for its reliable adhesion to skin for extended periods, while its orthotropic mechanical properties enable MT to measure strain unidirectionally along its longitudinal axis. Extensive lab tests have validated that MT exhibits a linear relationship between resistance and strain up to 10% peak tensile strains [29,30] and can be comfortably worn in both clinical and free-living environments [22]. By connecting MT to a custom, wireless, data acquisition node, skin-strain measurements can be streamed in real-time using Bluetooth Low Energy to a personal computer or smartphone.

Prior validation studies have investigated the accuracy of MT skin strain measurements against estimates from optical motion capture (mocap). During repeated squats, tensile and compressive skin strains measured on the tibialis anterior and gastrocnemius medialis showed strong correlations with mocap-estimated strain (ρ=0.96–0.98) [29]. Because mocap-estimated strain is computed from the normalized change in distance between two markers placed at the ends of the MT, it does not account for skin curvature. In contrast, MT measures strain along the skin surface underneath the sensing element and provides skin strain measurements with practically no time lag [29]. Preliminary studies have also demonstrated MT’s ability to measure localized skin strain associated with lumbar paraspinal muscle contraction [23], as well as its ability to distinguish between different low back movements in asymptomatic individuals [22]. Prior work has also employed a deep learning approach to classify lower back movements based on MT data [33] and biceps curl exercise movements [34]. MT’s non-rigid, skin-conforming design makes it well-suited for capturing localized strain patterns associated with the lumbar spine, which is a limitation of rigid sensors.

### 1.4. Open Questions

The feasibility of using MT strain data to distinguish individuals with LBP from healthy CTRLs, and to identify which movements, sensors, and biomechanical features are most informative for such discrimination, has not been systematically evaluated. Moreover, given recent interest in pretrained time-series foundation models (TSFMs) for learning generalizable representations across diverse temporal domains [35,36], it remains unclear whether such models can capture meaningful structure in MT-derived biomechanical strain, or whether task-specific feature engineering remains necessary.

In this work, we assessed whether MT strain time-series encode reliable group structure by training movement-specific ensemble classifiers on biomechanical features using a leave-pair-out (LPO) evaluation scheme. This is a uniquely challenging problem due to high participant inter-variability, movement-dependent biomechanics, and the absence of clear, task-invariant strain markers associated with LBP. In this exploratory study, we analyzed data from 20 participants performing 19 movements, each recorded from six MTs placed on the lumbar spine. Our study focused on identifying (i) which movements provided strong discriminative signals, (ii) which features were most informative and interpretable, and (iii) whether time-series encoders captured useful structure in this biomechanical sensing regime.

### 1.5. Contributions

1. We studied the feasibility of distinguishing participants with LBP from healthy CTRLs on a novel dataset using MT strain signals across a diverse set of 19 movements.
2. We showed that discriminative information was movement- and sensor-specific, with forward flexion and lower lumbar MTs providing the best signal for classification.
3. We demonstrated that movement-aware temporal features and specific feature combinations, rather than individual features or feature count, drove high performance.
4. We found that pretrained TSFMs did not transfer effectively to MT strain data, likely due to out-of-distribution mismatch with pretraining data and objectives.

## 2. Methodology

**Figure 1:**
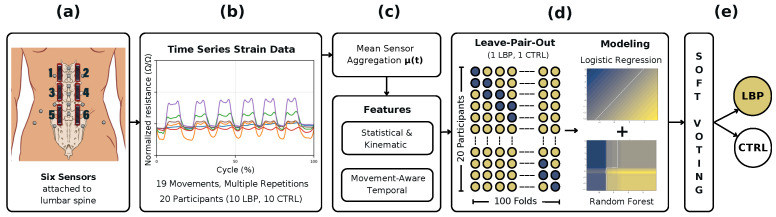
Overview of the full classification pipeline. (**a**) Six MT sensors were placed on the lumbar spine. (**b**) MT sensors recorded strain time-series during 19 functional movements with multiple repetitions across 20 participants (10 LBP, 10 CTRL). (**c**) Sensor channels were aggregated into a representative movement-specific strain signal, from which statistical/kinematic and movement-aware temporal features were extracted. (**d**) Logistic regression and random forest ensemble were trained under an LPO cross-validation protocol, where each fold held out one LBP and one CTRL participant. (**e**) Predictions from the two models were combined via soft voting to classify participants as LBP or CTRL.

Our objective was to distinguish individuals with LBP from healthy CTRLs using biomechanical strain signals captured by MT. Figure 1 provides an overview of the full modeling pipeline, from raw sensor data acquisition to final classification. Briefly, six MTs were placed in a 3 × 2 grid on the lumbar spine to record changes in strain over time during different functional movements. For each participant and movement type, these multichannel time-series were aggregated according to the sensor configuration being evaluated, producing a representative movement-specific strain signal.

Biomechanical features were then extracted from this signal to summarize strain magnitude, variability, loading rate, smoothness, and return-phase temporal dynamics. The statistical and kinematic features captured global markers of the full movement signal, whereas the movement-aware temporal features additionally captured peak-based and return-phase dynamics. These participant-level feature vectors were used to train a logistic regression (LR) [37] and random forest (RF) [38] ensemble under an LPO protocol, where each fold was trained on 18 participants and tested on one pair (CTRL and LBP), resulting in 90-10 splits and 100 folds with all possible combinations. The predictions from LR and RF were combined via soft voting to classify participants as LBP or CTRL.

### 2.1. MT Data

The dataset consisted of 20 participants (10 LBP, 10 CTRL), each of whom performed 19 distinct movements (see Appendix A for details of each movement), with multiple repetitions per movement. Inclusion criteria for the participants were defined as the following: (1) age between 18 and 65, (2) either no history of LBP in the last year or presence of chronic LBP (as defined by the NIH Research Task Force [39]), (3) ability to follow movement instructions in English or Spanish, and (4) capacity to perform simple trunk movements and functional activities like walking. Demographic characteristics and group composition of the cohort are summarized in Appendix B. Additional clinical characterization of the LBP group is summarized in Table 1 and Appendix C. Briefly, LBP duration ranged from 6 months to more than 5 years, and participants reported variable LBP frequency over the past 6 months, ranging from less than half the days to daily symptoms. Baseline pain (Standing) on the day of testing was also recorded using a 0–10 Numeric Pain Rating Scale (NPRS). These pain-related variables were reported only for cohort characterization and were not used as model inputs.

Six MTs were placed bilaterally along the lumbar spine in a 3 × 2 configuration which spans from the thoracolumbar junction to the lumbosacral junction (T12–S1), where T12 is the last thoracic vertebra, L1–L5 are the lumbar vertebrae, and S1 is the first sacral vertebra: the top MTs 1 and 2 were placed across T12 to L1 and L1 to L2 junctions, the middle MTs 3 and 4 were placed across the L2 to L3 and L3 to L4, and the bottom MTs 5 and 6 were placed across L4 to L5 and L5 to S1 (the placement is depicted on the left side of Figure 1). In addition, all kinesiology tapes were identical in size, and the piezoresistive sensing regions were manufactured at the same relative location on each tape. All sensors were placed by the same investigator (YV), who was trained by a physical therapist and biomechanist (SPG) to identify the relevant anatomical landmarks and place sensors. Sensor placement was standardized across participants. For lateral placement, sensors were placed just lateral to the spinous processes over the bulk of the erector spinae muscle bellies. For vertical placement, the investigator first palpated the L4 spinous process and positioned the bottom edge of the middle MT level just above L4 spinous process, spanning the L3–L4 intervertebral junction. The upper and lower MTs were then placed above and below the middle MT, directly adjacent to them with minimal vertical separation to avoid overlap.

Each MT measured local skin strain via changes in electrical resistance and was sampled at 60 Hz. MTs were fabricated following the procedure from [31] for multi-walled carbon nanotubes, which were shown to exceed the performance of graphene nanosheets from earlier studies in terms of consistency of signal stability. Raw resistance signals were normalized as

$$x_{s}\left(t\right)=\frac{R(t)}{R_{\mathrm{initial}}}-1$$

where $x_{s}\left(t\right)$ denotes the strain signal, R(t) is the resistance at time *t* (in Ohms, Ω), and $R_{\mathrm{initial}}$ denotes the baseline resistance recorded during a neutral (standing or sitting) posture prior to movement onset. Baseline resistance was measured prior to each movement type.

**Figure 2:**
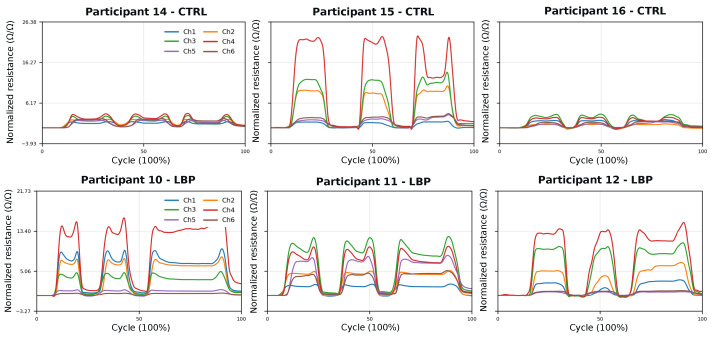
Representative MT strain across all MTs for different participants for forward flexion. Ch1–Ch6 correspond to the six MTs, ordered according to their placement along the lumbar spine.

Figure 2 provides representative visualizations of normalized resistance for the six MTs during the forward flexion movement across representative participants. The curves illustrate participant-specific variation in strain magnitude and temporal patterns, including primary-phase (baseline to peak strain) and return-phase (peak strain to baseline) behavior during movements.

All movements were repeated three times, except for continuous tasks such as driving (5 min), walking (35 feet), and stair climbing (10 stairs) (Appendix A). For activity-based movements, a repetition included both the task execution and the return component when applicable; for example, placing a tablet in a cabinet and retrieving it, picking up a suitcase and returning it, or screwing and unscrewing a lightbulb. For features computed over the full movement sequence, signals were processed across all repetitions without segmenting or averaging features separately per repetition. For features defined over the final repetition, the repetition boundary was identified using near-baseline strain levels before and after the peak strain time. Movement velocity or execution timing was not controlled for in the current study. Participants were allowed to move at a self-selected speed across movement tasks. This was an intentional methodological decision to ensure tasks were self-paced rather than externally paced from a motor control perspective and that individuals performed tasks as they typically would in a real-world environment. This also allowed for temporal variability characteristics to be explored with sensor-based measures. If a metronome or other form of pacing were used to control movement velocity or timing of tasks, it would result in an externally paced motor control task and alter movement parameters.

For each movement, signals from multiple MTs were combined into a single representative strain signal x(t). We systematically evaluated multiple sensor aggregation strategies, including single MT, selected MTs based on their locations (top, middle, bottom, left, or right sensors), and all six MTs. We also evaluated two fusion methods: feature-level and signal-level averaging. In signal-level averaging, selected raw MT signals were averaged first and features were then extracted from the aggregated signal. In feature-level averaging, features were extracted separately for each selected MT channel and then averaged across channels. We performed aggregation to reduce signal-level noise while preserving the dominant biomechanical signal associated with the task. We compared alternative sensor fusion strategies in Section 3.4 where we show that signal-level averaging outperforms feature-level averaging. Movements with missing sensor channels due to transient detachment were handled on a per-movement basis, which ensured that participants were retained while excluding unreliable measurements. All subsequent feature extraction and modeling were performed on x(t).

### 2.2. Feature Engineering

We defined two complementary feature sets from x(t) to compare the utility of different feature representations for classification, designed to capture discriminative biomechanical structure. Precise feature definitions, names, and interpretations are provided in Table 2.

Feature Set 1 (FS1) consists of commonly used global descriptors. We refer to these features as global because they are computed over the full movement duration without segmentation and summarize overall movement magnitude, variability, excursion, loading rate, and smoothness across the entire signal for a participant and movement type, rather than characterizing specific movement sub-phases. Specifically, this set includes peak strain (maximum absolute strain magnitude), strain variability (standard deviation of strain), Range of Motion (max–min strain excursion), max loading rate (maximum absolute first derivative), and smoothness (a jerk-based smoothness metric). These descriptors are motivated by prior biomechanics and movement-sensing studies showing that LBP is associated with altered range of motion, movement variability, trunk coordination, and movement smoothness [10,12,40,41]. These features capture coarse differences across participants and provide strong baseline representations for comparison with the movement-aware temporal features in Feature Set 2 (FS2).

FS2 is designed to probe finer-grained temporal structure that is not captured by global statistics alone. FS2 partially overlaps with FS1, retaining three features (peak strain, strain variability, and max loading rate) and extending with peak-based and return-focused features designed to analyze structure in the return phase of the movements. Specifically, FS2 includes post-peak trend (linear slope of strain following the peak), time near peak (duration spent above 95% of peak strain), post-peak irregularity (variability of gradient changes during return), time to half max (time required to reach 50% strain reduction), and end-task stability (strain variability during the final 25% of return). As shown in later analyses, these additional features were critical for resolving specific failure cases that persisted under FS1 alone.

All time-based features were computed relative to the peak strain time $t_{M}$. MT signals were sampled at 60 Hz, so each sample corresponds to 1/60 s, and temporal quantities are expressed as the number of samples following $t_{M}$ divided by 60. $t_{M}$ was defined over the repetition sequence and was identified either globally across all repetitions or within a task-specific repetition window (e.g., final repetition), depending on the feature and its intended interpretation.

### 2.3. Modeling and Evaluation Protocol

We formulated the task as binary classification between LBP and CTRL participants. For each movement, feature vectors were computed independently per participant. To ensure robust evaluation under the small sample size, we used an LPO cross-validation strategy. In each fold, one LBP and one CTRL participant were held out for testing, while the remaining 18 participants were used for training, which resulted in 100 folds. For our small cohort that was balanced with 10 participants with LBP and 10 matched CTRLs, we chose LPO rather than random 80/20 splits because LPO exhaustively evaluates all LBP–CTRL held-out pairs, ensuring that every participant was evaluated in the test set while reducing dependence on a particular random split and avoiding any data leakage from sample splits. Prior methodological work has shown that LPO can provide nearly unbiased AUC estimates with low variance in pairwise classification settings [42,43].

We employed two complementary classifiers—an LR model to capture linear separability and an RF to model nonlinear interactions—forming a simple model ensemble, with final predictions obtained via soft voting by averaging posterior probabilities. The RF model was trained with 200 trees, a maximum depth of 3, and minimum leaf and split sizes of 4, encouraging shallow decision structures and reducing overfitting. We also used an $\ell_{2}$-regularized logistic regression with C=0.1, optimized with the limited-memory Broyden–Fletcher–Goldfarb–Shanno (L-BFGS) solver and a maximum of 2000 iterations. A random state of 42 was used.

For completeness, we evaluated several alternative classifiers on the most discriminative configuration (forward flexion, MTs 4 and 6, FS2). The LR + RF ensemble had the highest performance in our setting (see Appendix D for classifier selection details). As a baseline, we also evaluated pretrained TSFMs (Chronos, MOMENT) [35,36] to assess whether general-purpose representations captured discriminative structure in MT strain signals.

Classification thresholds were selected using Youden’s J statistic on the training data within each fold, maximizing the trade-off between sensitivity and specificity and ensuring fair accuracy estimation [44]. Rather than pooling data across movements, we trained movement-specific classifiers. This design choice allowed us to identify which functional movements can best discriminate between the two groups.

Performance was evaluated using two complementary LPO accuracy measures. First, pooled LPO prediction-level accuracy was computed across all held-out predictions. Because each of the 100 LPO folds contained one held-out CTRL and one held-out LBP participant, this provided 200 potential held-out predictions, and accuracy was computed as the number of correctly classified held-out predictions divided by 200. Second, participant-level aggregated LPO accuracy was computed by averaging each participant’s held-out predicted probabilities across the folds in which that participant appeared as a test subject, yielding one aggregated probability score per participant. The final participant-level prediction was assigned as LBP if this aggregated probability score was greater than or equal to the classification threshold selected by Youden’s J statistic, and CTRL otherwise.

Misclassified participants reported in the results refer to participants whose final participant-level aggregated prediction was assigned the incorrect group label. Across movements, pooled LPO accuracy and participant-level aggregated LPO accuracy were strongly correlated (Pearson r=0.958, Spearman ρ=0.954), indicating that the two summaries ranked movement discriminability similarly.

All fold-sensitive procedures including feature normalization, classifier training, and threshold selection were nested within the cross-validation loop. Hyperparameters were fixed a priori. The feature sets FS1 and FS2 were defined prior to any analysis.

## 3. Results

We began by evaluating whether general-purpose TSFMs could capture discriminative structure in MT strain signals. This served as a baseline to assess whether off-the-shelf representations provided any discriminative signal for LBP. We then showed that our biomechanical features could help to successfully distinguish LBP from CTRL participants. Lastly, we identified the most discriminative movements, sensors, and feature combinations, and provided interpretable characterizations of group differences.

### 3.1. Weaknesses of Time-Series Embedding Analysis

**RQ1.** Do pretrained time-series foundation models capture biomechanical structure for LBP from MT signals?

Recent work has proposed pretrained TSFMs as general-purpose representations that can transfer across diverse temporal domains [35,36]. To assess whether such models capture participant-level biomechanical structure relevant to LBP in MT strain signals, we evaluated frozen MOMENT (base, large) and Chronos (base, large) encoders and tested whether their representations exhibited discriminative signal structure or sensitivity to controlled perturbations.

Embeddings were pooled over signals and averaged per participant to obtain participant-level representations. Analysis revealed extremely high cosine similarity across both intra-group (CTRL–CTRL, LBP–LBP) and inter-group (CTRL–LBP) comparisons, with similarities often approaching 0.99 as shown in Table 3. This near-indistinguishability suggested that the pretrained representations did not encode reliable group separation, making classifiers operating solely in embedding space ineffective; this was further confirmed by linear probing, which achieved only near-chance accuracy. Full details of the evaluation procedure, linear probing, and embedding perturbation protocol are provided in Appendix E.

**Figure 3:**
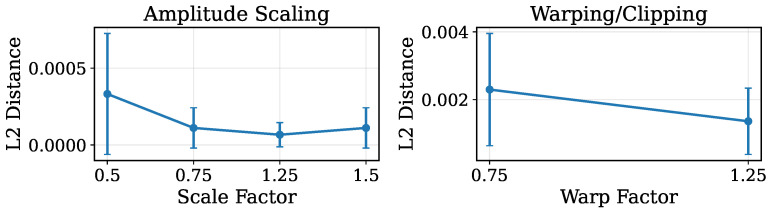
Sensitivity of pretrained embeddings to scaling and warping/clipping perturbations.

To further test the robustness of this observation, we applied two perturbations to the original time-series: amplitude scaling and temporal warping/clipping (Figure 3). For amplitude scaling, changing the overall signal magnitude led to only very small changes in the learned embeddings, with $\ell_{2}$ distance (Euclidean distance between embedding vectors) remaining close to zero across all tested scale factors.

Similarly, temporal warping/clipping (compression and stretching) induced very minor embedding shifts, indicating that the representations are largely invariant to these temporal distortions. These results suggest that MT biomechanical signals constitute an out-of-distribution regime for current pretrained time-series models, likely because these models were trained primarily on large-scale forecasting datasets that differ substantially from localized, movement-driven MT strain dynamics.

Finally, to verify that our embedding evaluation pipeline was sensitive to standard time-series structure, we applied the same protocol to three datasets from the University of California, Riverside (UCR) Time Series Classification Archive [45] (electric devices, earthquakes, and electrocardiograms). This validation was included to distinguish between a general failure of the embedding pipeline and a domain-specific failure on MT strain signals. The pipeline produced meaningful separation and perturbation sensitivity on the UCR datasets, supporting the interpretation that the failure is specific to MT signals rather than the evaluation procedure itself (see Appendix E.2).

The takeaway was that general-purpose time-series models did not meaningfully capture LBP-relevant structure in MT strain data. This negative result motivated our next set of experiments, where we used features that explicitly encode temporal dynamics.

### 3.2. Classification of LBP vs. CTRL Using Biomechanical Features

**RQ2.** Which movements reliably distinguish LBP and CTRL participants?

#### 3.2.1. FS1 Baseline Performance

We first briefly summarized classification performance for movements using FS1. Forward flexion emerged as the strongest task under this set, where the ensemble achieved 85% accuracy on the test folds (95% confidence interval: 64.0–94.8%). However, consistent misclassifications were observed across several movements. Within forward flexion, errors persisted for CTRL participants 15 and 18 and LBP participant 22, indicating that strain magnitude statistics alone did not fully capture individualized patterns. Full FS1 results across all movements and MT pairs are provided and discussed in Appendix F. To evaluate whether additional structure could resolve such outliers, we next augmented the feature set with return-phase and peak-based temporal descriptors (FS2).

#### 3.2.2. FS2 Performance and Movement Discrimination

Accuracy varied substantially across movements with FS2 as shown in Table 4, with clear differences in their discriminative utility. With FS2, forward flexion once again emerged as the strongest single task under both evaluation summaries, achieving 95% (95% CI: 76.4–99.1%) participant-level aggregated LPO accuracy (19/20 participants) and 87.0% pooled LPO prediction-level accuracy (174/200 held-out predictions). The pooled LPO result indicated that forward flexion remained the most reliable movement even when performance was evaluated across all held-out fold predictions, while the participant-level aggregated result summarized the final subject-level classification after averaging each participants’ held-out probabilities across LPO folds. This improvement resolved nearly all prior failure cases with FS1 in the sample set for forward flexion, with CTRL participant 18 and LBP participant 22 now correctly classified. Only CTRL participant 15 remained misclassified at the participant level. These gains were attributable to improved feature expressivity. Temporal features captured biomechanical differences in the strain return phase of the movement that were not reflected in global statistics alone.

In contrast to forward flexion, the other movements revealed important limitations and failure modes. Several movements, including DriveSit and Extend, retained moderate discriminative power but exhibited both false positives and false negatives. DriveSit achieved 85% participant-level aggregated accuracy and 78.5% pooled LPO accuracy, whereas Extend achieved 75% participant-level aggregated accuracy and 66.5% pooled LPO accuracy. Misclassifications in these tasks were distributed across multiple participants rather than concentrated in a single outlier, indicating weaker and less stable biomechanical signals compared to forward flexion. A larger group of movements, such as BottleLeft, Lightbulb, TabletFloor, RevRight, and other reaching or carrying tasks, clustered around 60–70% participant-level aggregated accuracy and showed similarly moderate pooled LPO performance.

Several movements, including LROT, RROT, WeightedSuitcaseLeft, and WeightedSuitcaseRight, collapsed to chance-level performance at 50%. In these cases, nearly all LBP participants were misclassified, showing substantial overlap in feature space between groups and limited differentiation, possibly due to the lack of discriminative structure between the two groups. We observed that across the other movements, most notably CTRL participants 15, 18, and 9 and LBP participants 6, 5, and 3, recurred among misclassifications.

We also noted that combining features across multiple movements degraded performance rather than improving it. Forward flexion alone achieved 95% participant-level aggregated LPO accuracy, whereas fusing it with DriveSit or BottleLeft reduced accuracy to 75%, and fusion with Extend reduced it further to 60%. This pattern indicated that less discriminating movements actively dilute the strong localized signal present in forward flexion, and that movement-specific classification is preferable to indiscriminate pooling across tasks.

Importantly, the persistence of CTRL participant 15 as a failure case supported its interpretation as a genuine biomechanical outlier. One possible explanation is that their movement pattern reflected individual-specific biomechanical variability that overlaps with patterns observed in the LBP group. An additional possibility is that this participant may present early biomechanical changes consistent with LBP. However, confirming any future clinical presentation of LBP would require prospective investigation and is beyond the scope of this study.

#### 3.2.3. Feature Set Tradeoff: FS1 vs. FS2

Extending FS1 with the temporal return-phase and peak-based descriptors in FS2 introduced an important tradeoff. While FS2 enabled improvements in several cases by resolving difficult participant-level outliers, its effects were not uniformly positive across all movements. In best-case MT configurations, FS2 yielded notable gains for some tasks (e.g., +15% on WeightedSuitcaseRight and +10% on Lightbulb), but also led to degradations for others (e.g., −10% on TabletFloor).

The tradeoff between FS1 and FS2 was instructive for forward flexion as shown in Table 5, where FS2 improved accuracy for several sensor configurations (most notably from 85% to 95% on the optimal pair 4, 6) but also caused degradations in others (e.g., from 85% to 80% on the four-sensor set 3, 4, 5, 6). We therefore view FS1 as a strong and broadly viable baseline representation, with FS2 serving as a refinement that is useful when additional temporal structure is needed to address specific failure cases.

#### 3.2.4. Permutation Test and Participant Consistency

To assess whether the 95% accuracy of forward flexion could arise from chance label structure, we performed a permutation test [46] with 1000 runs. In each run, participant labels (LBP/CTRL) were randomly shuffled, and the full LPO evaluation pipeline was reapplied to forward flexion (MTs 4 and 6, FS2). We observed that no permuted run achieved 95% accuracy; the maximum observed was 70%, and the empirical null distribution centered at 50% (p<0.001). This confirmed that the observed discriminative structure reflected genuine biomechanical differences rather than chance. Across the 100 LPO test folds for the same setting, we also observed that 18 out of 20 participants were correctly classified in more than 80% of folds where they appeared as test subjects. Only CTRL 15 and LBP 6 showed lower consistency. This pattern argued against results being driven by idiosyncratic subject-level outliers; instead, most participants contributed reliably to the discriminative signal.

#### 3.2.5. Feature Count and Subset Analysis

**Figure A3:**
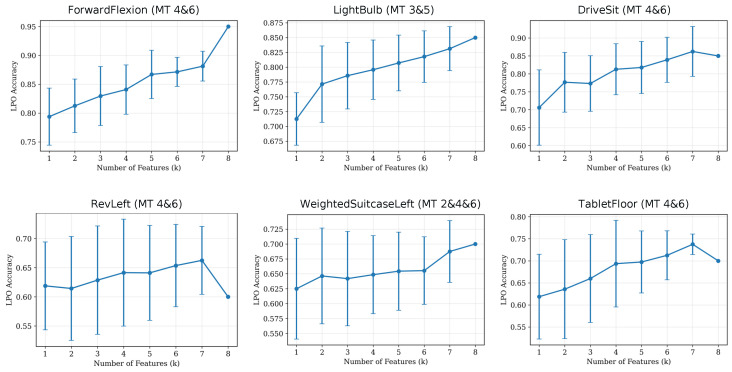
LPO test accuracy as a function of feature set size for multiple movements and MT configurations.

We further analyzed the classification performance across movements and MT pair configurations as a function of feature count using an exhaustive post hoc subset evaluation as a robustness check for feature composition analysis, showing that accuracy generally increased with additional features but in a strongly movement-dependent and non-monotonic manner (see Appendix G, Figure A3). For high discriminative movements, performance increased steadily when more features were added, whereas low discriminative movements showed no consistent gain beyond a small number of features. Thus, there was no feature count that was optimal across movement types.

**Figure A4:**
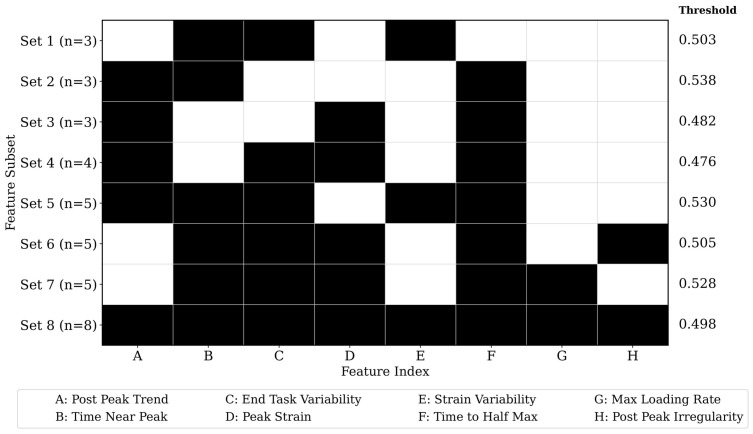
Feature–Set Incidence Matrix for 95% Accuracy Subsets (forward flexion). Black indicates feature inclusion in the subset; white indicates absence.

Furthermore, we examined which feature combinations achieved high accuracy and found that no single feature was sufficient; instead, performance arose from multiple combinations of features rather than a fixed feature set (see Appendix G, Figure A4). The classification accuracy depended on movement-specific feature design. Specifically, for forward flexion we identified eight distinct feature subsets that achieved 95% test accuracy, with subset sizes ranging from three features to the full set of eight. The optimal classification threshold varied across these subsets, which indicated that the decision boundary was sensitive to which biomechanical features were included. Notably in this experiment, Lightbulb was the only other movement that approached high performance, reaching 90% accuracy across approximately 8 feature subsets.

### 3.3. Feature Characterization

**RQ3.** What biomechanical feature-level differences distinguish LBP and CTRL participants?

**Figure 4:**
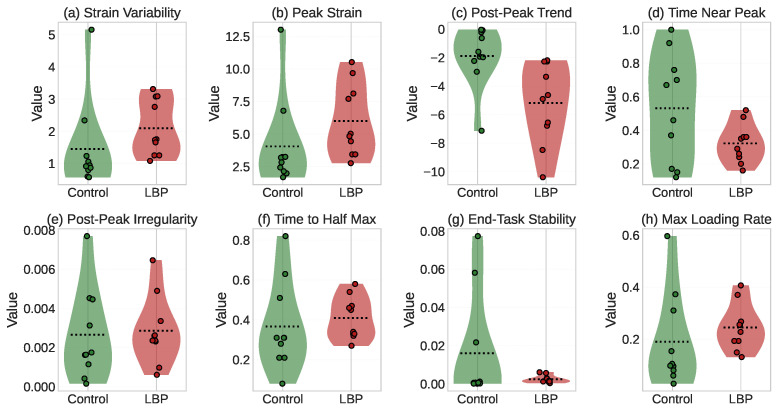
Distribution of features (FS2) for participants with CTRL and LBP during forward flexion.

Following the identification of forward flexion as the most discriminative movement, we examined feature-level distributions for this task to provide interpretability into the biomechanical characteristics captured by the engineered features as shown in Figure 4. Each subplot shows participant-level feature values with overlaid individual data points for direct comparison of central tendency, spread, and overlap between groups.

Across features, we observed distributional differences between participants with LBP and CTRLs, with participants with LBP generally showing higher strain magnitudes and loading rates, and CTRLs showing greater inter-subject variability across several features. These visualizations are intended to aid interpretability of the engineered features rather than to imply clear separability in the learned decision space. The observed differences are reflected across several strain features. Importantly, no single feature serves as a dominant marker, and discriminative power arises from their combination. These distributional differences and feature patterns which we summarize below are broadly consistent with frameworks of altered motor control in people with LBP [9,11,12,13].

(a) **Strain variability** was generally higher in participants with LBP which indicated increased fluctuation in strain during the movement.
(b) **Peak strain** values tend to be higher for participants with LBP, suggesting that they experienced larger maximal strain magnitudes.
(c) **Post-peak trend** showed that participants with LBP exhibited more negative return-phase slopes, indicating a faster decline in strain immediately after the peak.
(d) **Time near peak** was generally lower for participants with LBP. CTRLs showed higher and more variable plateau durations, indicating that they were able to hold near-maximal bend positions for a longer period of time.
(e) **Post-peak irregularity** exhibited mixed low and high values in both groups. This indicated that return-phase smoothness varied across individuals in both participants with and without LBP.
(f) **Time to half max** was generally higher in participants with LBP, although CTRL participants also show notable variability, indicating that return-phase speed differs across individuals but is more often slowed in participants with LBP.
(g) **End-task stability** showed no clear group separation, with most participants in both groups converging to low residual variability by the end.
(h) **Max loading rate** was generally higher in participants with LBP, indicating more abrupt strain increases during movement execution.

**Figure 5:**
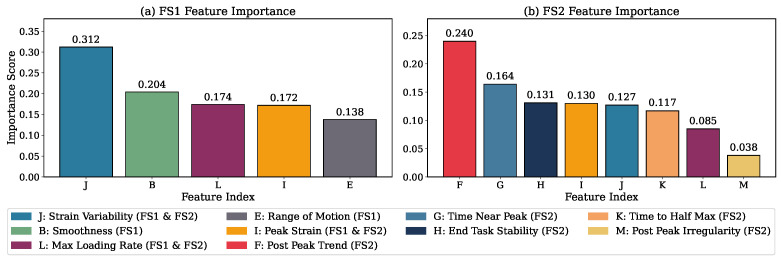
Feature importance comparison for the forward flexion movement. (**a**) Importance scores for global magnitude features (FS1). (**b**) Importance scores for temporal features (FS2).

We further examined feature importance to assess how FS1 and FS2 differ in the information they provide for forward flexion classification. Importance scores were derived from the ensemble model by combining RF impurity-based importance and absolute LR coefficients, computed per fold and averaged across cross-validation folds before being combined into a final score. As shown in Figure 5, importance in FS1 was concentrated on global magnitude and variability measures, with Strain Variability contributing most. In contrast, FS2 shifts importance toward temporal return-phase features, led by post peak trend and time near peak, while shared magnitude-based features play a reduced but consistent role.

### 3.4. MT Placement and Fusion Analysis

**RQ4.** How does sensor placement and fusion affect classification performance?

Because MTs capture localized strain at different anatomical locations, it was unclear a priori which MTs best encode discriminative structure for LBP. We therefore systematically evaluated how sensor placement and sensor fusion strategy influence classification performance, with the goal of identifying minimal yet informative sensing configurations.

#### 3.4.1. Sensor Placement

**Figure 6:**
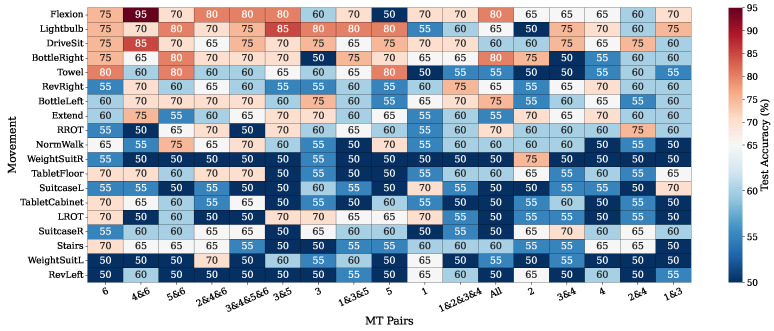
Test-set accuracy across movements under varying MT configurations for FS2. Rows correspond to individual movements, and columns denote single MT, MT pairs, and the full six-MT configuration. Rows are sorted by the highest accuracy achieved for that movement (best-performing movement at the top). Columns are sorted by the mean accuracy of each sensor configuration (best average performance on the left).

We analyzed classification behavior as shown in Figure 6 using FS2 under systematically varied sensor configurations, including (i) single-sensor models using one MT channel at a time, (ii) multi-sensor combinations of varying size (e.g., 2–4 MTs), and (iii) aggregation across all six MTs, to examine how sensor placement influences the extracted signal. FS1 results are provided in Appendix F.

Across movements, sensor contributions were clearly non-uniform. MTs located in the lower lumbar spine, particularly 4 and 6 (right side), consistently captured more discriminative structure than MTs positioned more superiorly. MTs 4 and 6 lie adjacent to the paraspinal regions in the middle to lower lumbar region and crossing the lumbosacral junction, which experience substantial coordinated strain during and return from forward flexion. This spatial pattern indicates that biomechanical differences associated with LBP are not expressed uniformly.

While certain individual MTs, most notably MT 6, remained informative for classification even when used in isolation, single-sensor configurations generally exhibited weaker and less consistent performance across movements. So, although strong localized signals did exist, spatial context was often necessary to fully characterize the multi-axial strain and dynamics associated with LBP.

Pairwise MT configurations further clarified the role of spatial coupling. Combining spatially adjacent or bilaterally symmetric MTs substantially improved signal coherence, particularly for MT pairs spanning both sides of the lower lumbar spine. These configurations captured complementary strain patterns that reflect coordinated primary-phase and return-phase behavior, highlighting that relative strain evolution across regions conveys discriminative information. Importantly, the benefit of sensor pairing was movement dependent. For movements like forward flexion, a small subset of lower sensors was sufficient to capture the dominant biomechanical signature. In contrast, movements involving mixed or asymmetric primary-phase behavior exhibit more diffuse and unstable sensor relevance, leading to reduced and less consistent performance.

Finally, using all six sensors did not consistently improve classification performance beyond that achieved by the most informative MT subsets. In several movements, full six MT aggregation yielded comparable or even degraded performance relative to targeted pairings, suggesting that indiscriminate spatial aggregation can introduce noise or attenuate localized signals when weaker sensors contribute minimally informative or inconsistent strain patterns. Importantly, because the most informative MT pairings vary by movement, we believe maintaining coverage across all sensor locations was necessary to ensure robust discrimination across the full set of functional movements. The best-performing MT pair configurations for each movement under FS1 and FS2 are summarized in Appendix H.

#### 3.4.2. Ablation on Sensor Fusion Strategies

Next, we further compared two sensor fusion strategies using the most informative sensor pair (MTs 4 and 6) for the forward flexion movement. Feature-level averaging computed features for each sensor individually and then averaged the resulting feature vectors, whereas signal-level averaging first averaged the raw strain signals across all MTs and then extracted features from the aggregated time-series.

**Figure 7:**
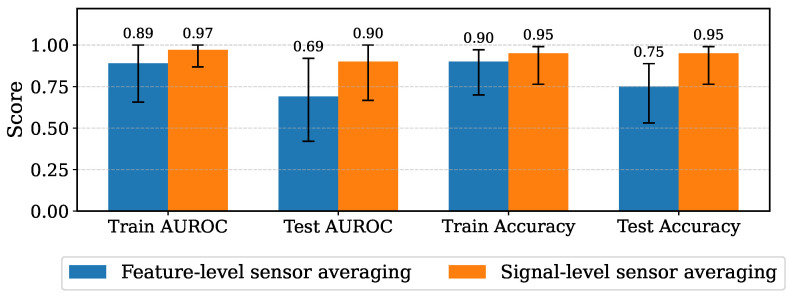
Comparison of sensor fusion at feature-level (features computed per sensor and then averaged) vs signal-level (averaged sensor signals, then features computed from aggregates) strategies using MTs 4 and 6 for forward flexion. Values shown above the bars indicate the reported metric values; error bars indicate 95% confidence intervals, with bootstrap intervals used for AUROC and Wilson intervals used for accuracy.

Signal-level averaging showed higher performance across all metrics as depicted in Figure 7. Test area under the receiver operating characteristic curve (AUROC) increased from 0.69 to 0.90, and test accuracy improved from 0.75 to 0.95 for signal-level compared to feature-level averaging, with similar improvements observed on the training set. These results indicated that aggregating sensor signals prior to feature extraction provided better discriminative representations across sensors. We therefore used signal-level averaging for all analyses in this study.

## 4. Discussion

This exploratory study evaluated whether MT strain signals encode discriminative structure for distinguishing individuals with LBP from healthy CTRL participants. We view the present study as identifying (i) which movements, sensors, and features were most discriminative, (ii) methodological best practices for MT-based classification including sensor fusion strategies, modeling, and feature set design, (iii) evidence that current TSFMs did not transfer to this biomechanical regime, and (iv) a reproducible evaluation framework for future MT studies.

From a clinical perspective, the discriminative power of forward flexion was consistent with existing LBP biomechanics literature. Prior studies comparing clinical populations with LBP to CTRLs have reported group differences in movement-based parameters during forward flexion and return from forward flexion [12,26,47,48,49,50]. The return phase of forward flexion, captured by our temporal features such as post-peak trend, time near peak, and time to half max, may reflect clinically relevant differences in trunk movement behavior. These findings were broadly consistent with prior work showing that pain and LBP can be associated with altered motor control, although the specific presentation of these changes may vary across individuals and tasks [9,11].

The finding that pretrained TSFMs did not meaningfully capture LBP-relevant structure in MT strain data was consistent with a broader pattern of limitations observed across time-series models, large language models, and vision-language models on structured temporal data across various tasks and evaluation settings [51,52,53,54,55,56,57].

The findings also have direct implications for integrating MT sensing into clinical practice. Rather than requiring all sensor locations and movement tasks, our results suggest that a reduced sensor configuration and movement set may still provide useful biomechanical information while simplifying data collection. As the importance of sensors and movements varies across classification scenarios, the optimal configuration should be application-specific and validated in larger cohorts, improving the practicality and feasibility of MT-based assessments while preserving informative biomechanical readouts.

The primary limitation of this study was the sample size of 20 participants, which constrained statistical power for generalization. We note that MT is a novel custom-fabricated sensor for which no external datasets exist, and larger human-subject collection was constrained by hardware availability, and the need for supervised laboratory acquisition. This study is explicitly framed as an exploratory study: its goal was to determine whether MT signals encode discriminative structure for LBP, and to identify which movements, sensors, and features warrant prioritization in larger-scale validation and not to produce population-level performance estimates. In addition, because our goal was not to validate an existing movement-based clinical classification system, we did not use clinical measures for participant selection or to validate our MT measures. Further, future work should evaluate whether MT strain measurements can identify movement-based LBP subgroups. Such studies will require a heterogeneous sample of people with LBP to capture variability in movement characteristics and MT strain measures. We also acknowledge that people without LBP can display movement variability, and thus it is important to identify features that distinguish people with LBP from CTRLs despite this variability. These processes may be useful in future studies that include larger prospective cohorts for diagnostic purposes.

A cohort of 20 participants is consistent with, and in many cases larger than, analogous published studies of novel wearable sensing technologies at equivalent stages of development. Representative examples include: gait analysis using triboelectric smart socks (N=13) [58], wearable fall detection (N=10) [59], multimodal wearable sensing for dementia detection (N=17) [60], wearable strain sensor measurement of respiratory rate (N=20) [61], and recent exploratory works studying immersive rehabilitation with wearable sensors (N≤20) [62,63]. In each of these cases, small cohort sizes were accepted as appropriate for the exploratory scope of the study.

However, the small number of participants and the feature-to-participant ratio may raise concerns regarding overfitting, but we take a rigorous approach to mitigate this. The design features of the present study provided methodological safeguards against overfitting and chance-level results within the current study. First, each participant contributed data across 19 functional movements and 6 sensor channels, resulting in a structurally rich dataset with multiple task repetitions for each movement. Second, holding out a test set that the model never sees during training is a standard approach to address overfitting; our LPO protocol ensures that the model was tested on unseen data and, by exhausting all LBP–CTRL participant pairs, ensures that performance estimates were not driven by a single held-out split. Third, the finding that forward flexion achieved high accuracy while several other movements collapse to chance level constitutes an internal negative control. If results reflected idiosyncratic subject-level patterns rather than genuine biomechanical structure, we would not expect such selective movement-dependent discrimination. This collapse to chance level also argues against trivial participant-level confounds. Fourth, bootstrap stability analysis (Appendix I) showed that discriminative features remain consistently significant as participants are progressively added. Fifth, a 1000-run label-shuffled permutation test confirmed that the observed 95% accuracy for forward flexion is not a chance artifact (p<0.001), with no permuted run reaching this level. Finally, 18 out of 20 participants were correctly classified in more than 80% of their test folds, arguing against results being driven by a small number of outliers.

The validity of LPO cross-validation in small-*N* settings is supported by prior methodological work. Prior work [42,43] has demonstrated that LPO produces nearly unbiased AUC estimates with low variance compared to alternatives such as *k*-fold cross-validation and showed that LPO estimators remain reliable in small-sample regimes where other estimators become unstable. Importantly, LPO ensures that every participant pair was evaluated as a test set, minimizing evaluation variance given the available cohort.

We believe the scale of this study was appropriate for its stated goals. We acknowledge that the absence of an independent validation cohort remains a key limitation of this exploratory study and validation in independent, larger cohorts is essential before clinical deployment, and we frame all conclusions accordingly. As an exploratory analysis and because no prior MT-based LBP effect size estimates were available for an a priori power analysis, we quantified the magnitude of group-level separation for forward flexion features using standardized effect sizes computed between participants with LBP and CTRLs. Post-peak trend exhibited the largest separation (Cohen’s d=−1.33), followed by time near peak (d=−0.86) [64]. Based on these observed effects, a future validation study would require approximately N≈45 participants per group to achieve 80% power to detect the median observed effect at α=0.05. To account for potential effect size attenuation in independent cohorts, we conservatively recommend N≥70 participants per group for definitive validation. Therefore, the present cohort is underpowered for definitive clinical conclusions, and the classification results should be interpreted only as exploratory evidence of discriminative MT signal structure rather than validated diagnostic performance.

The LBP cohort in this exploratory study was defined using NIH Research Task Force criteria, but detailed clinical phenotyping was limited. Although pain-related descriptors collected during the movement protocol are reported in Appendix C, additional variables characterizing their clinical presentation such as disability measures, psychosocial measures, medication use, and physical activity level were not collected. Because biomechanical behavior may differ substantially across clinical profiles, the present results should not be interpreted as representative of the population of all patients with LBP. Larger studies with more variability and richer clinical characterization are needed to evaluate whether MT-based discriminative patterns vary across clinical presentations.

The participant age range was broad (18–65 years), which could introduce age-related variability in movement characteristics. Age was not included as a covariate in the classification models because of the small cohort size and the risk of overfitting. However, each participant in the CTRL group was age-matched (±5 years) to a participant in the LBP group (CTRL age: 41.8±15.7 years; LBP age: 42.0±16.7 years), which reduces the likelihood that age alone explains the observed group differences.

## 5. Conclusions

In this exploratory study, we showed that MT strain signals can provide objective and interpretable markers for distinguishing participants with LBP from CTRL participants across functional movements. Discriminative biomechanical structure was not globally expressed, but instead emerged from specific interactions between movement type, sensor placement, and temporal strain dynamics. Forward flexion uniquely supported stable, high-accuracy classification, driven by coordinated patterns of strain magnitude, load persistence, and post-peak return-phase rather than any single feature. Temporal dynamics were important for resolving participants that global statistics fail to separate. Informative signals were spatially localized to the lower lumbar spine, with selective sensor pairing outperforming indiscriminate aggregation. In contrast, pretrained time-series models failed to capture participant-level structure in MT signals and remained largely invariant to simple perturbations, indicating a mismatch with localized biomechanical sensing. Overall, our results provide preliminary evidence that MT is a promising and interpretable sensing modality for conducting movement-based assessment in people with LBP.

## Figures and Tables

**Table 1:** Clinical pain-related descriptors for participants with LBP. Participant IDs correspond to the original study IDs.

Participant ID	LBP Duration (in Years (yr))	LBP Frequency	Baseline Pain (Standing)
3	1 ≤ yr < 5	Daily/nearly daily	1
5	0.5 ≤ yr < 1	≥half days	4
6	1 ≤ yr < 5	Daily/nearly daily	6
7	>5 yr	Daily/nearly daily	4
10	>5 yr	Daily/nearly daily	0
11	1 ≤ yr < 5	≥half days	0
12	1 ≤ yr < 5	<half days	1
13	>5 yr	≥half days	5
19	>5 yr	≥half days	1
22	>5 yr	<half days	2

**Table 2:** Names, definitions, and interpretation of features. Feature set membership is indicated by colored squares: FS1, FS2, and shared features (FS1/FS2). Notation: $x_{t}\equiv x\left(t\right)$ is the aggregated strain signal, $\nabla x_{t}$ = discrete gradient, $\Delta x_{t}=x_{t+1}-x_{t}$ is first difference, $f_{s}=60$ Hz (sampling rate), #{·} = sample counts, *k* = integer offset after peak strain time $t_{M}$, *T* = number of time samples.

Set	Feature Name	Definition	Interpretation
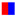	Peak Strain	$M=\max_{t}\left|x_{t}\right|$	Highest strain magnitude reached
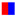	Strain Variability	$\sigma =\mathrm{std}(x_{t})$	Overall variability in strain during movement
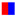	Max Loading Rate	$L_{\max}=\max_{t}\left|\nabla x_{t}\right|$	Maximum instantaneous strain change
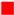	Smoothness	$J=-\log\;\left(\frac{1}{T}\sum_{t=1}^{T}{\left(\nabla^{3}x_{t}\right)}^{2}+\varepsilon \right)$	Measure of motion smoothness based on jerk magnitude (ε is small constant)
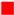	Range of Motion	$R=\max_{t}\left(x_{t}\right)-\min_{t}\left(x_{t}\right)$	Difference between maximum and minimum strain
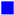	Time Near Peak	$\tau =\frac{\#\{t:\;|x_{t}|\geq 0.95M\}}{f_{s}}$	Total duration spent at or above 95% of maximum strain
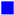	Post-Peak Trend	$\beta =\mathrm{slope}({\left\{x_{t}\right\}}_{t\geq t_{M}})$	Linear trend of strain over the return phase (last repetition)
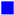	Post-Peak Irregularity	$I={\mathrm{mean}}_{t\geq t_{M}}\;\left(\left|\nabla x_{t}-\nabla x_{t-1}\right|\right)$	Average change in strain slope during return phase (last repetition)
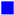	Time to Half Max	$\tau_{50}=\frac{\min\{k:\;|x_{t_{M}+k}|\leq 0.5M\}}{f_{s}}$	Time from peak to first 50% strain reduction
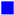	End-Task Stability	$S={\mathrm{std}}_{t\in R_{\mathrm{end}}}\;\left(\Delta x_{t}\right)$	Variability in strain changes over final 25% of post-peak segment ($R_{\mathrm{end}}$) (last repetition)

**Table 3:** Intra-group and inter-group cosine similarity (mean ± standard deviation) for time-series encoders. Separation score is the difference between within-group and between-group similarity.

Model	CTRL–CTRL	LBP–LBP	Between-Group	Separation Score
MOMENT-base	0.9963±0.0033	0.9974±0.0019	0.9970±0.0030	−0.0001
MOMENT-large	0.9922±0.0059	0.9907±0.0068	0.9907±0.0088	0.0008
Chronos-base	0.9737±0.0195	0.9883±0.0059	0.9821±0.0128	−0.0011
Chronos-large	0.9616±0.0271	0.9792±0.0104	0.9716±0.0186	−0.0012

**Table 4:** Movement-wise performance on FS2 ordered by participant-level aggregated LPO accuracy, with ties sorted by pooled LPO accuracy. Results are based on MT pair 4 and 6; the rationale for this selection is discussed in Section 3.4.2. Participant-level aggregated accuracy was computed after averaging each participants’ held-out predicted probabilities across LPO folds, yielding one final prediction per participant. Pooled LPO accuracy was computed across all 200 held-out predictions from the 100 LPO folds. Misclassified CTRL/LBP entries list participant IDs based on the participant-level aggregated predictions.

Movement	Participant-Level Accuracy (%)	Pooled LPO Accuracy (%)	Misclassified CTRL	Misclassified LBP
ForwardFlexion	95 (19/20)	87.0 (174/200)	15	–
DriveSit	85 (17/20)	78.5 (157/200)	9, 18	5
Extend	75 (15/20)	66.5 (133/200)	14, 20	3, 7, 10
TabletFloor	70 (14/20)	69.5 (139/200)	9, 15, 18, 20	6, 22
Lightbulb	70 (14/20)	68.5 (137/200)	9, 14, 15, 16	3, 11
BottleLeft	70 (14/20)	67.0 (134/200)	15, 18, 20, 21	10, 11
RevRight	70 (14/20)	66.5 (133/200)	8, 9, 15, 18, 20	12
BottleRight	65 (13/20)	57.5 (115/200)	9, 15, 18, 20, 21	6, 10
TabletCabinet	65 (13/20)	57.5 (115/200)	9, 14, 15, 18, 20	5, 6
Stairs	65 (13/20)	57.0 (114/200)	9, 14, 15	3, 6, 7, 19
RevLeft	60 (12/20)	56.0 (112/200)	1, 8, 9, 14, 18, 20	6, 12
Towel	60 (12/20)	53.5 (107/200)	9, 14, 15	3, 5, 6, 10, 22
SuitcaseRight	60 (12/20)	50.5 (101/200)	9, 14, 16, 18	5, 6, 19, 22
NormWalk	55 (11/20)	50.5 (101/200)	1, 9, 15, 16, 20	3, 6, 10, 19
SuitcaseLeft	55 (11/20)	45.5 (91/200)	1, 15, 18	3, 5, 6, 7, 12, 13
LROT	50 (10/20)	50.0 (100/200)	–	3, 5, 6, 7, 10, 11, 12, 13, 19, 22
RROT	50 (10/20)	50.0 (100/200)	–	3, 5, 6, 7, 10, 11, 12, 13, 19, 22
WeightedSuitcaseLeft	50 (10/20)	50.0 (100/200)	–	3, 5, 6, 7, 10, 11, 12, 13, 19, 22
WeightedSuitcaseRight	50 (10/20)	50.0 (100/200)	–	3, 5, 6, 7, 10, 11, 12, 13, 19, 22

**Table 5:** Forward Flexion accuracy (%) for each sensor configuration under FS1 and FS2. Δ denotes the change in accuracy computed as FS2 − FS1, where green values indicate improvement and red values indicate degradation.

Config	All	Four Sensors		Three Sensors		Two Sensors						Single Sensors					
	**All**	**1, 2, 3, 4**	**3, 4, 5, 6**	**1, 3, 5**	**2, 4, 6**	**1, 3**	**2, 4**	**3, 4**	**3, 5**	**4, 6**	**5, 6**	**1**	**2**	**3**	**4**	**5**	**6**
FS1	85	65	85	65	75	70	65	70	65	85	70	55	70	65	65	65	70
FS2	80	70	80	70	80	70	60	65	80	95	70	70	65	60	65	50	75
** Δ **	−5	+5	−5	+5	+5	0	−5	−5	+15	+10	0	+15	−5	−5	0	−15	+5

**Figure A1:**
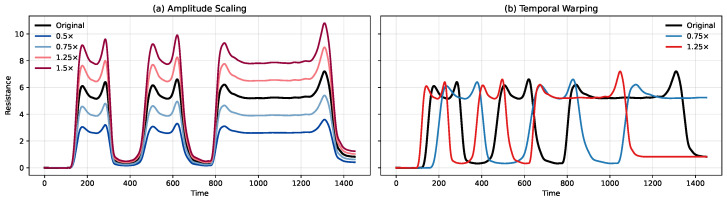
Representative example of amplitude scaling and warping/clipping during forward flexion. Y-axis represents normalized resistance (Ω/Ω).

**Figure A2:**
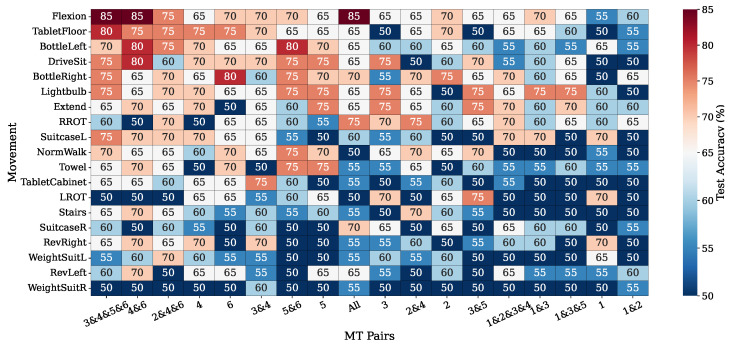
Test-set accuracy across movements under varying MT configurations for FS1. Rows correspond to individual movements, and columns denote single MT, MT pairs, and the full six-MT configuration.

**Figure A5:**
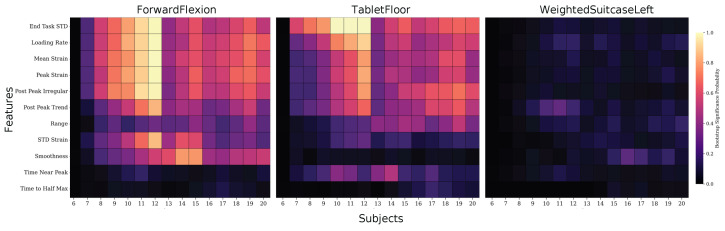
Bootstrap significance stability for three representative movements selected based on their classification performance. Forward flexion (high), TabletFloor (moderate), and WeightedSuitcaseLeft (weak) performing movement. Heatmaps show the probability that each feature is significant as the number of included participants increases.

## Data Availability

The de-identified dataset used in this study is available upon appropriate request to the corresponding author. The data are not publicly available due to ethical concerns.

## Appendix A. Movement Details

Table A1 summarizes the functional movements performed by participants, along with their task descriptions and execution protocols. Throughout the paper, we refer to movements using the acronyms listed in parentheses for brevity.

**Table A1:** Movements movements performed by participants and their corresponding descriptions.

Movement	Position	Direction	Task Description
**Clinical Movement Tasks**			
Forward Flexion	Standing	Flexion	Bending forward halfway the first two times and then as far as possible the third time.
Extension (Extend)	Standing	Extension	With hands supporting at the hips, bending the shoulders backward over the hips as far as possible without losing balance.
Rotation (left, right) (LROT, RROT)	Sitting	Rotation	Rotate the shoulders left (right) as far as possible, keeping the hips in place.
**Driving Tasks**			
Driving (DriveSit)	Sitting	Flexion	Sitting on the seat of a golf cart like when driving, with both feet firmly on the ground.
Reverse Driving (left, right) (RevLeft, RevRight)	Sitting	Rotation	Sitting on the seat of a golf cart, rotate the shoulders backward to the left (right) as if driving a car and going in reverse, then return to facing forward.
**Pickup Functional Tasks**			
Picking up tablet from floor (TabletFloor)	Standing	Flexion	Bending forward to place a tablet on the floor and then return to upright; instructed to use the back and hips but may bend slightly in the knees if needed.
Placing tablet in cabinet (TabletCabinet)	Standing	Flexion	Bending forward to place a tablet in a lower cabinet, return to upright, then bend forward to retrieve the tablet from the cabinet and return to upright.
Picking up bottle (left, right) (BottleLeft, BottleRight)	Sitting	Lateral Bending	Bending to the left (right) to pick up a water bottle on the floor with your left (right) hand without holding the chair, return to upright, then return the bottle to the floor using the same movement and return to upright.
Picking up suitcase (left, right) (SuitcaseLeft, SuitcaseRight)	Standing	Lateral Bending	Bending to the left (right) to pick up the suitcase with your left (right) hand, return to upright, then return the suitcase to the floor using the same movement and return to upright.
Picking up weighted suitcase (left, right) (WeightedSuitcaseLeft, WeightedSuitcaseRight)	Standing	Lateral Bending	See description above. Suitcase is weighted with a 10-pound weight.
**Additional Functional Tasks**			
Hanging a towel (Towel)	Standing	Extension	Stand with feet a comfortable distance apart, holding a towel with both hands. Reach overhead to hang the towel on a hook, return arms to side, then reach overhead to remove the towel from the hook and return arms to side.
Putting in lightbulb (Lightbulb)	Standing	Extension	Stand with feet a comfortable distance apart directly under the light. Reach upward with full back extension to screw a bulb into the socket, return to a resting position, then repeat by unscrewing the bulb and returning to rest.
Stair Climbing (up and down) (Stairs)	Standing	N/A	Walking up 10 stairs to the top the staircase, pause and turn around, then walk down the same 10 steps; may lightly touch the handrail for balance if needed.
Normal walking (NormWalk)	Standing	N/A	Walking on a flat straight sidewalk at a normal pace. Walk from one marked line to another in a straight direction and return to rest.

## Appendix B. Demographic Data

In this section, we summarize the composition and demographic characteristics of the study cohort in Table A2 and Table A3. Participant group membership was balanced across CTRL and LBP cohorts. The two groups were closely matched in age, height, weight, and sex assigned at birth, reducing the likelihood that observed classification differences are driven by demographic confounds rather than biomechanical factors.

**Table A2:** Group membership of participants included in the final analysis cohort.

Group	Participant Identifiers
LBP	3, 5, 6, 7, 10, 11, 12, 13, 19, 22
CTRL	1, 8, 9, 14, 15, 16, 17, 18, 20, 21

**Table A3:** Aggregated demographic characteristics of participants included in the analysis cohort.

Variable	CTRL	LBP
Number of participants	10	10
Age (years), mean ± SD	41.8±15.7	42.0±16.7
Age range (years)	20–64	22–65
Sex assigned at birth (F/M)	5/5	6/4
Height (cm), mean ± SD	171.8±13.5	172.0±7.3
Weight (kg), mean ± SD	78.8±12.8	80.7±23.2

The LBP cohort in this study represents individuals with clinically reported low back pain rather than a single uniform diagnosis. As expected in real-world clinical populations, participants differed in symptom chronicity, severity, and underlying etiology, reflecting the natural diversity of LBP profiles. In this work, our primary objective was to distinguish participants with LBP from CTRLs using movement-specific strain signatures, rather than to model or separate clinical subtypes. Identifying subtype-specific biomechanical patterns within LBP is an important and promising direction for future research but is beyond the scope of the current cohort and study design.

## Appendix C. Pain Data

Pain data are reported in Table A4 and Table A5 to provide additional clinical characterization of the cohort. Baseline pain denotes the participant’s NPRS pain rating on the day of testing. For forward flexion, pre pain and post pain denote pain ratings before and after the task, respectively. Pain direction denotes the participant-reported direction of pain change: no pain (NP), no change (NC), increase (INC), or decrease (DEC). Pain change is computed as post-task pain minus pre-task pain.

**Table A4:** Summary of pain characteristics for participants with LBP. Baseline pain denotes NPRS pain rating on the day of testing. Participant IDs correspond to the original study IDs.

Pain Characteristics	N = 10 (%)
LBP Frequency	
Daily/nearly daily	4 (40%)
≥half days	4 (40%)
<half days	2 (20%)
LBP Duration	
6 mo–1 yr	1 (10%)
1–5 yr	4 (40%)
>5 yr	5 (50%)
Baseline Pain	Median 1.5; range 0–6

These pain-related variables were intentionally excluded from the main classification analysis. The goal of the study was to evaluate whether MT strain signals alone encode biomechanical structure that distinguishes participants with LBP from CTRL participants. Including pain-related variables as model inputs would directly encode clinical symptom information and could confound the evaluation of MT strain signals as a biomechanical sensing modality. Although LBP duration and frequency are reported for cohort characterization, other clinical descriptors, including disability measures, radiculopathy status, medication use, and physical activity level, were not collected in this study. As a result, we cannot determine whether the MT-based classification patterns are associated with specific clinical characteristics or subgroups within LBP.

**Table A5:** Pain-related cohort descriptors for the forward flexion task.

Participant	Group	Baseline Pain	Forward Flexion			
			**Pain Direction**	**Pre Pain**	**Post Pain**	**Pain Change**
1	CTRL	0	NP	0	0	0
3	LBP	1	INC	1	4	3
5	LBP	4	INC	4	5	1
6	LBP	6	NC	6	6	0
7	LBP	4	INC	5	6	1
8	CTRL	0	NP	0	0	0
9	CTRL	0	NP	0	0	0
10	LBP	0	NP	0	0	0
11	LBP	0	INC	0	2	2
12	LBP	1	NC	1	1	0
13	LBP	5	DEC	4	3	-1
14	CTRL	0	NP	0	0	0
15	CTRL	0	NP	0	0	0
16	CTRL	0	NP	0	0	0
17	CTRL	0	INC	0	2	2
18	CTRL	0	NP	0	0	0
19	LBP	1	INC	2	4	2
20	CTRL	0	NP	0	0	0
21	CTRL	0	NP	0	0	0
22	LBP	2	INC	2	3	1

## Appendix D. Classifier Comparison

To justify the choice of LR+RF ensemble over more complex models given the small cohort, we evaluated alternative classifiers on the most discriminative configuration (forward flexion, MTs 4 and 6, FS2) under the same LPO protocol. Results are summarized in Table A6. More flexible models did not improve generalization and showed signs of overfitting. The simple ensemble of linear and tree-based models proved most appropriate for this interpretability-focused setting.

**Table A6:** Comparison of alternative classifiers on forward flexion (MTs 4 and 6, FS2).

Classifier	Test Accuracy (%)	95% CI (Wilson)
XGBoost [65]	65	[0.433, 0.819]
CatBoost [66]	65	[0.433, 0.819]
RBF-SVM [67]	70	[0.481, 0.855]
2-layer MLP	50	[0.299, 0.701]
LR + SVM ensemble	70	[0.481, 0.855]
**LR + RF (ours)**	**95**	**[0.764, 0.991]**

## Appendix E. Embedding Experiment Setup

In this section, we describe the experimental setup used to evaluate the representations of MOMENT and Chronos models for MT strain signals, which includes perturbation-based sensitivity analysis and validation on other time-series classification datasets.

### Appendix E.1. Probing Embedding Sensitivity via Controlled Perturbations

We evaluated the sensitivity of time-series embeddings to controlled perturbations applied directly to MT strain. To test sensitivity to changes in signal magnitude, we applied multiplicative amplitude scaling to the averaged strain signal:

$$x^{\left(s\right)}\left(t\right)=s\cdot x\left(t\right),\;s\in \left\{0.5,0.75,1.25,1.5\right\}.$$

Scaled signals retained the original temporal structure while modifying the overall strain magnitude.

To test sensitivity to temporal distortions, we applied uniform time warping/clipping. Given a factor *w*, the signal was defined as

$$x^{\left(w\right)}\left(t\right)\;=\;x\;\left(\phi_{w}\left(t\right)\right),$$

$$\phi_{w}\left(t\right)\;=\;\min\left(wt,\;1\right),\;t\in \left[0,1\right],$$

where x(·) is evaluated via linear interpolation. This procedure preserves the original signal length by resampling onto a fixed grid and clipping boundary values. We evaluated compression (w=1.25) and stretching (w=0.75). An illustrative example of how the perturbations are applied are shown in Figure A1.

Each perturbed signal was passed through a frozen pretrained time-series encoder. For each perturbation, we computed the $\ell_{2}$ distance between the perturbed embedding and the embedding of the original signal. Distances were aggregated across all movements to obtain mean and standard deviation statistics. For amplitude scaling, results were grouped by scale factor. Mean and standard deviation of embedding distances were computed across movements and visualized using error bars. An analogous procedure was used for temporal warping/clipping experiments. Notably, fairly aggressive temporal distortions for warping/clipping induced minimal embedding displacement, suggesting that representations were largely insensitive to the localized dynamics in MT strain.

To further assess whether pretrained embeddings encode participant-level group structure, we performed a linear probing experiment. We extracted frozen participant-level embeddings from MOMENT-base by pooling over time. We then trained an LR classifier as a linear probe using the leave-pair-out protocol. Linear probe performance remained near chance (55% accuracy), indicating that even supervised linear readouts cannot reliably separate the two groups in embedding space. To verify that this failure is not an artifact of the linearity of the readout, we additionally trained a 2-layer MLP on the same frozen embeddings under the same LPO protocol; performance remained near chance (Chronos: 55%, MOMENT: 50%), confirming that the limitation was representational rather than a consequence of readout expressivity. We also note that finetuning on domain-specific MT data could potentially improve transfer; however, with only 10 samples per class, this would be statistically inappropriate at the current cohort scale.

### Appendix E.2. Validation of the Embedding Analysis Pipeline on University of California Riverside Time Series Datasets

To confirm that our embedding sensitivity analysis pipeline is functioning correctly and that the failure on MT data is meaningful, we applied the same protocol to three UCR time-series classification datasets [45]: ElectricDevices, Earthquakes and ECG5000. For each dataset, we (i) randomly selected 300 samples from two classes to match MT cohort size, (ii) extracted frozen MOMENT-large embeddings, (iii) computed intra-class and inter-class cosine similarities, and (iv) measured embedding $\ell_{2}$ distance after 0.75× temporal warping. Table A7 summarizes the results.

**Table A7:** Comparison of embedding behavior on UCR datasets vs. MT data.

Dataset	Class A-A	Class B-B	Between-Class	Separation Score	Warp (0.75×) $\ell_{2}$
ElectricDevices	0.7941	0.7216	0.6930	0.0649	1.4518
Earthquakes	0.9213	0.9425	0.9191	0.0128	0.9141
ECG5000	0.8536	0.8172	0.7982	0.0372	1.3951
MT (ours)	0.9922	0.9907	0.9907	0.0008	0.0022

For UCR datasets, embeddings showed meaningful within-class vs. between-class separation (positive separation scores) and substantial sensitivity to temporal warping ($\ell_{2}$ distances >0.9). In contrast, MT embeddings showed near-perfect uniformity across all comparisons and near-zero sensitivity to warping. This confirmed that the embedding pipeline was sound; the failure was specific to MT biomechanical strain signals, which lie outside the distribution these foundation models were trained to represent.

## Appendix F. Feature Set 1 Full Results

Test-set accuracy across movements and MT configurations under FS1 shows substantial variability across both tasks and sensor locations (Figure A2). Forward flexion consistently achieved the highest accuracy, particularly when using MT pairs spanning the lower lumbar region, whereas many other movements cluster near chance-level performance, indicating limited discriminative power of magnitude-based features alone.

An important open question is whether more specialized, movement-specific biomechanical descriptors could be developed for the near-chance tasks, where the current feature representations may fail to capture subtler or qualitatively different strain dynamics. Another promising direction is to identify task-specific feature subsets from within our existing full feature sets, which may better isolate the discriminative structure present in these movements. These remain important and interesting directions for future work.

## Appendix G. Ablations on Feature Combinations

### Appendix G.1. Feature Count Analysis

To examine how classification performance depends on feature composition, we performed an exhaustive feature subset analysis using the eight interpretable biomechanical features defined in FS2. This analysis was post hoc and supplementary to the main results. For each movement and MT configuration, we evaluated all possible non-empty subsets of these features, giving 255 total subsets spanning feature counts from k=1 to k=8. For each subset size k, we computed LPO test accuracy independently for every subset of that size. Accuracy was evaluated using the same ensemble classifier and cross-validation protocol used throughout the paper. Results are summarized as the mean LPO accuracy across all subsets of size k, with variability reflecting sensitivity to feature selection (Figure A3).

We observed that accuracy generally tends to improve as more features are included, but the relationship was not monotonic and was strongly movement dependent. For highly discriminative movements such as forward flexion, performance increased steadily and peaks when all features are used, indicating that multiple complementary descriptors jointly capture the underlying biomechanical signal.

In contrast, weaker movements (e.g., RevLeft) saturated early or show no consistent gains beyond a small number of features, reflecting limited or unstable discriminative structure. Variability across subsets is highest for small feature counts, where performance depends strongly on which specific features are selected. As feature count increases, variance decreases, which shows robustness, although additional features can introduce redundancy for some movements.

These results also further suggest that no single feature count was universally optimal. Instead, discriminative performance depended on the movement type, MT sensor configuration, and feature composition, highlighting the need for movement-specific feature design.

### Appendix G.2. High-Performing Feature Subsets

The accuracy–feature count analysis showed that while higher performance is often achieved with larger feature sets, feature count alone did not explain which features drive discrimination. Examining subsets that reach 95% accuracy revealed that no single feature was sufficient, even for forward flexion. Instead, high performance arose from specific combinations of features.

For forward flexion, we identified 8 distinct feature subsets that achieve 95% test accuracy, spanning subset sizes from 3-feature sets up to the full 8-feature set (Figure A4). We used Youden’s *J* statistic to determine the optimal classification threshold for each subset. The resulting thresholds varied across feature subsets, indicating that decision boundary is sensitive to the specific biomechanical descriptors included. For the full 8-feature configuration (Set 8), the optimal threshold is 0.498.

No other movement which we tested for the subset analysis attains this accuracy level under exhaustive subset evaluation. Among the forward flexion subsets, no single feature is universally present; instead, high accuracy emerged from different feature combinations, explaining why smaller subsets can match the performance of larger ones. Notably, Lightbulb movement was the only other task to approach high performance, reaching 90% accuracy across approximately 8 distinct feature subsets.

## Appendix H. Best MT Pairs for Each Movement

Table A8 summarizes the best-performing MT sensor combinations identified for each movement under the two feature sets (FS1 and FS2). For each movement, we report the MT pair that provided the highest inter-group discrimination accuracy (LBP vs. CTRL) under the LPO evaluation protocol. MT pair 4 and 6 consistently emerged as the strongest-performing configuration across a wide range of movements, but best-performing configurations varied based on movement.

**Table A8:** Best-performing MT pairs for each movement under feature sets FS1 and FS2.

Movement	FS1	FS2
ForwardFlexion	4, 6	4, 6
TabletFloor	4, 6	4, 6
BottleLeft	4, 6	4, 6
DriveSit	4, 6	4, 6
Lightbulb	4, 6	3, 5
Extend	4, 6	4, 6
BottleRight	4, 6	5, 6
RROT	2, 4	2, 4
SuitcaseLeft	4, 6	4, 6
NormWalk	5, 6	5, 6
Towel	5, 6	5, 6
TabletCabinet	3, 4	4, 6
LROT	3, 5	3, 5
Stairs	4, 6	4, 6
SuitcaseRight	1, 2, 3, 4	3, 4
RevRight	4, 6	4, 6
WeightedSuitcaseLeft	2, 4, 6	2, 4, 6
RevLeft	4, 6	4, 6
WeightedSuitcaseRight	2	2

## Appendix I. Feature Stability

In this section, we evaluated the stability of biomechanical features using a progressive bootstrap significance analysis to assess whether discriminative signals persist as additional participants are included. This analysis was performed for all movements and their features; results for selected movements are shown in Figure A5. For each movement and feature, participants were incrementally added from N=6 to N=20. At each *N*, 500 bootstrap resamples were performed, and group differences between LBP and CTRL participants were tested using a two-sided Mann–Whitney U test (α = 0.05). Feature stability was defined as the fraction of bootstrap runs in which a feature is significant, giving a value between 0 and 1.

To illustrate the range of observed stability patterns across movements, we highlight three representative tasks. Forward flexion exhibited a robust pattern, with most of the features reaching high and persistent significance probabilities as N increases. Global magnitude and temporal features remained stable across participant inclusion which indicated cohort-robust discriminative structure. TabletFloor showed qualitatively similar but weaker significance compared to forward flexion, with many features attaining moderate values only at intermediate N and failing to persist. WeightedSuitcaseLeft demonstrated uniformly low significance across all features and participant counts. This behavior was also representative of other low-performing movements and indicated that such tasks did not encode reliable biomechanical differences between LBP and CTRL participants under the proposed feature representation.
